# Supplementary material for: Characterising the HIV self-testing market in Kenya: Awareness and usage, barriers and motivators to uptake, and propensity to pay
Source: PLOS Glob Public Health. 2023 Apr 5;3(4):e0001776. doi: 10.1371/journal.pgph.0001776 (PMC10075389; doi:10.1371/journal.pgph.0001776)
Supplement: S1 Appendix — (DOCX) [file pgph.0001776.s002.docx]

**S1 Appendix. Target and achieved sample**

| NAIROBI | | | | | | | | | | |
| --- | --- | --- | --- | --- | --- | --- | --- | --- | --- | --- |
|  | **Total population** | | **Population estimate removing HIV-positive individuals based on HIV prevalence** | | **Population estimate removing PrEP users**  (mid-range estimate 4000 users) | | **Sample quota** | | **Final achieved sample** | |
|  | Male | Female | Male  (4.7% prevalence) | Female  (7.5% prevalence) | Male | Female | Male | Female | Male | Female |
| Aged 18-19 | 72,842 | 73,238 | 69,419 | 67,746 | 69,254 | 67,585 | 35 | 34 | 35 | 31 |
| Aged 20-29 | 571,729 | 574,838 | 544,858 | 531,725 | 543,566 | 530,465 | 274 | 268 | 274 | 282 |
| Aged 30-35 | 251,695 | 253,064 | 239,866 | 234,084 | 239,297 | 233,530 | 121 | 118 | 121 | 124 |

| KISUMU | | | | | | | | | | |
| --- | --- | --- | --- | --- | --- | --- | --- | --- | --- | --- |
|  | **Total population** | | **Population estimate removing HIV-positive individuals based on HIV prevalence** | | **Population estimate removing PrEP users**  (mid-range estimate 4000 users) | | **Final sample quota** | | **Final achieved sample** | |
|  | Male | Female | Male  (15.0% prevalence) | Female  (17.4% prevalence) | Male | Female | Male | Female | Male | Female |
| Aged 18-19 | 29,044 | 30,815 | 24,688 | 25,453 | 24,362 | 25,117 | 12 | 13 | 13 | 14 |
| Aged 20-29 | 101,766 | 107,970 | 86,501 | 89,183 | 85,358 | 88,005 | 43 | 44 | 44 | 45 |
| Aged 30-35 | 44,547 | 47,272 | 37,865 | 39,930 | 37,364 | 38,523 | 19 | 19 | 20 | 20 |
