# Supplementary material for: Characterising the HIV self-testing market in Kenya: Awareness and usage, barriers and motivators to uptake, and propensity to pay
Source: PLOS Glob Public Health. 2023 Apr 5;3(4):e0001776. doi: 10.1371/journal.pgph.0001776 (PMC10075389; doi:10.1371/journal.pgph.0001776)
Supplement: S2 Appendix — (DOCX) [file pgph.0001776.s003.docx]

**S2 Appendix. Demographics by segment**

|  | **Total sample n = 1,021** | | | | |
| --- | --- | --- | --- | --- | --- |
|  | **Segment 1**  **n = 277** | **Segment 2**  **n = 242** | **Segment 3**  **n = 140** | **Segment 4**  **n = 185** | **Segment 5**  **n = 177** |
| **County** |  |  |  |  |  |
| Nairobi | 236 (85.2%) | 190 (78.5%) | 131 (93.6%) | 157 (84.9%) | 151 (85.3%) |
| Kisumu | 41 (14.8%) | 52 (21.5%) | 9 (6.4%) | 28 (15.1%) | 26 (14.7%) |
| **Gender** |  |  |  |  |  |
| Male | 147 (53.1%) | 119 (49.2%) | 63 (45.0%) | 91 (49.2%) | 85 (48.0%) |
| Female | 130 (46.9%) | 123 (50.8%) | 77 (55.0%) | 94 (50.8%) | 92 (52.0%) |
| **Age** |  |  |  |  |  |
| 18-24 | 102 (36.8%) | 99 (40.9%) | 64 (45.7%) | 92 (49.7%) | 82 (46.3%) |
| 25-30 | 105 (37.9%) | 93 (38.4%) | 42 (30%) | 59 (31.9%) | 54 (30.5%) |
| 31-35 | 70 (25.3%) | 50 (20.7%) | 34 (24.3%) | 34 (18.4%) | 41 (23.2%) |
| **Setting** |  |  |  |  |  |
| Urban | 240 (86.7%) | 188 (77.7%) | 112 (80.0%) | 158 (85.4%) | 149 (84.2%) |
| Peri-urban | 30 (10.8%) | 39 (16.1%) | 25 (17.9%) | 25 (13.5%) | 25 (14.1%) |
| Rural | 7 (2.5%) | 15 (6.2%) | 3 (2.1%) | 2 (1.1%) | 3 (1.7%) |
| **Main source of Income** |  |  |  |  |  |
| Parent/relative support | 26 (9.4%) | 34 (14.0%) | 25 (17.9%) | 32 (17.3%) | 24 (13.6%) |
| Farming | 0 (0.0%) | 0 (0.0%) | 1 (0.1%) | 0 (0.0%) | 2 (0.2%) |
| Private sector | 49 (17.7%) | 30 (12.4%) | 18 (12.9%) | 19 (10.3%) | 14 (0.8%)* |
| Civil service/government | 8 (2.9%) | 7 (2.9%) | 8 (5.7%) | 3 (1.6%) | 7 (4.0%) |
| Spousal support | 5 (1.8%) | 6 (2.5%) | 1 (0.1%) | 5 (2.7%) | 10 (5.6%) |
| Casual work | 42 (15.2%) | 34 (14.0%) | 19 (13.6%) | 27 (14.6%) | 24 (13.6%) |
| Domestic work | 7 (2.5%) | 6 (2.5%) | 3 (2.1%) | 5 (2.7%) | 5 (2.8%) |
| Informal sector | 18 (6.5%) | 11 (4.5%) | 10 (7.1%) | 14 (7.6%) | 17 (9.6%) |
| Other | 9 (3.2%) | 7 (2.9%) | 8 (5.7%) | 4 (2 2%) | 5 (2.8%) |
| **Education Level** |  |  |  |  |  |
| Never attended school | 1 (0.0%) | 0 (0.0%) | 1 (0.07%) | 1 (0.05%) | 3 (1.7%) |
| Primary | 30 (10.8%) | 25 (10.3%) | 9 (6.4%) | 18 (9.8%) | 32 (18.1%) |
| Secondary/A Level | 109 (39.4%) | 97 (40.1%) | 58 (41.4%) | 63 (34.1%) | 77 (43.5%) |
| College | 92 (33.2%) | 94 (38.8%) | 40 (28.6% | 65 (35.1%) | 38 (21.5%) |
| University | 45 (16.2%) | 26 (10.7%) | 32 (22.9%) | 38 (20.5%) | 27 (15.3%) |
| **Money earned in a year (KSH)** |  |  |  |  |  |
| Less than 10,000 | 38 (13.7%) | 52 (21.5%) | 19 (13.6%) | 38 (20.5%) | 36 (20.3%) |
| 10,001-30,000 | 34 (12.3%) | 43 (17.8%) | 28 (20.0%) | 31 (16.8%) | 28 (15.8%) |
| 30,001-50,000 | 26 (9.4%) | 26 (10.7%) | 14 (10.0%) | 22 (11.9%) | 17 (9.6%) |
| 50,001-70,000 | 33 (11.9%) | 18 (7.4%) | 16 (11.4%) | 16 (8.6%) | 26 (14.7%) |
| 70,001-90,000 | 36 (13.0%) | 19 (7.9%) | 7 (5.0%) | 23 (12.4%) | 19 (10.7%) |
| 90,001-110,000 | 34 (12.3%) | 27 (11.2%) | 14 (10.0%) | 22 (11.9%) | 18 (10.2%) |
| 110,001 and above | 71 (25.6%) | 52 (21.5%) | 41 (29.3%) | 32 (17.3%) | 33 (18.6%) |

* p<.05 ** p<.01 *** p<.001
