# Supplementary material for: Characterising the HIV self-testing market in Kenya: Awareness and usage, barriers and motivators to uptake, and propensity to pay
Source: PLOS Glob Public Health. 2023 Apr 5;3(4):e0001776. doi: 10.1371/journal.pgph.0001776 (PMC10075389; doi:10.1371/journal.pgph.0001776)
Supplement: Attachment — Submitted filename: Response to Reviewers.docx [file pgph.0001776.s004.docx]

**Response to Academic Editor**

1. Please send a completed 'Competing Interests' statement, including any COIs declared by your co-authors. If you have no competing interests to declare, please state "The authors have declared that no competing interests exist". Otherwise please declare all competing interests beginning with the statement "I have read the journal's policy and the authors of this manuscript have the following competing interests:"

***Response:*** *Thank for noting this, I have reviewed the Competing Interest policy and have updated the competing interest statement as follows:* The authors have declared that no competing interests exist.

2. Please amend your detailed Financial Disclosure statement. This is published with the article. It must therefore be completed in full sentences and contain the exact wording you wish to be published.

a. State the initials, alongside each funding source, of each author to receive each grant.
b. State what role the funders took in the study. If the funders had no role in your study, please state: “The funders had no role in study design, data collection and analysis, decision to publish, or preparation of the manuscript.”
c. If any authors received a salary from any of your funders, please state which authors and which funders.

If you did not receive any funding for this study, please simply state: “The authors received no specific funding for this work.”

***Response:*** *Thank you for providing this instruction; we have amended the funding statement as follows:* HA received funding for this research from the Children’s Investment Fund Foundation, UNITAID, and the Elton John AIDS Foundation. The funders had no role in study design, data collection and analysis, decision to publish, or preparation of the manuscript.

3. Please provide separate figure files in .tif or .eps format only and remove any figures embedded in your manuscript file. Please also ensure that all files are under our size limit of 10MB.

For more information about figure files please see our guidelines:

<https://journals.plos.org/globalpublichealth/s/figures> 
<https://journals.plos.org/globalpublichealth/s/figures#loc-file-requirement>

***Response:*** *Thank you, we have removed Figure 1 from the manuscript and uploaded it as a .tif file with the resubmission.*

4. We noticed that you used "data not shown and unpublished data" in the manuscript. We do not allow these references, as the PLOS data access policy requires that all data be either published with the manuscript or made available in a publicly accessible database. Please amend the supplementary material to include the referenced data or remove the references.

***Response:*** *Thank you, we have removed the references to the related data throughout the manuscript.*

5. Please amend your Data Availability Statement and indicate where the data may be found

***Response:*** *We have included the data as a Supporting Information in our resubmission and noted this in the data availability statement.*

**Reviewers’ Comments to the Author:**

Reviewer #1: I think the topic is of great interest but the rationale for the approach is not clear as currently written. A few things would increase enthusiasm for this paper

***Response:*** *Thank you for taking the time to review our paper and provide feedback. We will address your points in turn below.*

- a description of household level clustering of sampling and how that was accounted for

Response: Thank you for this question. We have added the following detail: *“**Interviews were carried out by Population Services Kenya* *in the 17 sub-counties of Nairobi and 2 sub-counties of Kisumu in which the Challenge Fund project was being implemented. Face-to-face interviews were conducted at the household level in participants’ homes in February 2022 using computer assisted personal interviewing via an online data capture platform (SurveyCTO) on smartphones and tablets. Interviewers randomly selected households using the following approaches: first, a central location point (usually a landmark) was identified in each village visited. Using the left hand rule approach, the first household was randomly selected using the date score (combining the two digits of the date, e.g., if research was carried out on the 12^th^, the sum of the two digits of the date was done (1+2=3) and the total sum 3 was taken as the random number for the first household). The interviewers would then use this random number to identify which house to start interviews (e.g., the 3^rd^ house to the left of the starting point). Interviewers would ask if anyone from the target age (18-35) was present; if yes, they would start the screening process. After a successful interview, interviewers would skip 4 households and attempt an interview in the 5^th^ house keeping the left hand rule, and so forth. If there were no eligible participants in the household, interviewers would proceed to the next house until a successful interview was completed; and then subsequently apply the 5^th^ household rule until the quota for that village was met or the day ended.”*

- refusal rate for enrollment

***Response:*** We did not collect data on refusals. This has been addressed in the discussion section as a limitation using the following language: “*Another limitation is that we did not collect data on number and nature of refusals, so we cannot comment on the nature of people opting out of this research and how it may have influenced our results.”*

We did collect data for reasons for screen-out. The following language has been added to the results section: *“A total of n=1021 individuals were interviewed; 62 were screened out for various reasons: being under age (n=35), not agreeing to consent (n=3), currently on ART or PrEP (n=11) and being HIV-positive (n=11).”*

- description of KSH to USD or some other currency for comparison

***Response:*** Thank you for noting this, we have included USD equivalents throughout the manuscript for ease of interpretation using a 10:1 KSH:USD ratio which was the exchange rate during the period of research.

- why the segmental analysis was pursued and why this is a helpful way to look at the data

***Response:*** Thank you for this comment. We realise that our description of segmentation could be greatly developed and have added the following text in the introduction: *“To investigate “consideration,” we conducted an attitudinal segmentation was used to identify and quantify unique groups of potential HIVST users and tease apart their unique barriers and facilitators to testing. Segmentation is an analytic tool borrowed for social and commercial marketing which is increasingly being used to tackle public health challenges, including HIV [18,19], voluntary male medical circumcision [20], family planning [21], and tuberculosis [22]. Segmentations can be used for developing targeted health information, programmes and messages for discrete sub-groups of a population that are similar in attitudes and behaviour [23–25], rather than relying on demographics (like age, gender) to develop public health interventions and communications. In the case of HIVST, a segmentation can be used to quantify groups within a population who are already using HIVST (e.g., early adopters), who are unlikely to ever use HIVST, and identify key drivers and barriers to use for potential users.*

- why PrEP users were excluded

***Response:*** The rationale for excluding PrEP users was that they were more likely to be	clinic-based HIV testing as part of routine PrEP provision and therefore not using HIVST. However, we acknowledge in hindsight that PrEP users may be an important group of HIVST users in the future, particularly if PrEP guidelines allow for HIVST results to be brought to the clinic rather than conducting clinic-based testing for follow-up visitsl or simply for self-monitoring whilst between clinic vists. We have noted this in the limitation sections: *Lastly, we excluded PrEP users from this sample as our assumption was that they would be more likely to access clinic-based HIV testing as part of routine PrEP provision; however, they may be an important group of HIVST users in the long-term as they can employ HIVST for self-care in between visits for PrEP. Additional research should examine the interest of PrEP users in utilising HIVST in Kenya.*

- would hvae been interested in seeting the data for the whole group on some of the varaibels collected like motivations and barriers to HIVST use,

***Response:*** Thank you for this comment. We have added these data into Tables 3 and 4 so the reader can compare the overall sample data to responses from the segments. However please note that for significance testing we have conducted ANOVA between segments. We have indicated this beneath the tables so that readers do not interpret significance test results as testing against the overall sample mean.

Reviewer #2: On the whole, this is a well-designed and implemented study. Perhaps what is a little unclear is exactly how were respondents to the study recruited. It is difficult to understand where the interviewers located the respondents. Was it at their houses? How were the interviews done i.e., were they paper based or electronic capture. Was itself administered interviews or face-to-face? Were there any declines? Just some precision in the description needed.

***Response:*** Thank you for taking the time to review this manuscript and provide your feedback. We have reorganised the methods section with additional sub-headers (survey design, sample, data collection, etc.) to try to make this part of the manuscript easier to read and follow overall, as well as addressing your specific comments (addressed below).

Regarding interview procedures, could we please draw your attention to the text on Page 6 where we state: *“Face-to-face interviews were conducted in participants’ homes in February 2022 using computer assisted personal interviewing via online data capture platform (SurveyCTO) on smartphones and tablets.” We hope that having this text under the newly labelled “Data collection” section makes it easier for the reader to navigate to find this information.*

In response to Reviewer 1’s comments about recruitment , we have also added the following detail regarding recruitment: *“Interviews were carried out by Population Services Kenya in the 17 sub-counties of Nairobi and 2 sub-counties of Kisumu in which the Challenge Fund project was being implemented. Face-to-face interviews were conducted at the household level in participants’ homes in February 2022 using computer assisted personal interviewing via an online data capture platform (SurveyCTO) on smartphones and tablets. Interviewers randomly selected households using the following approaches: first, a central location point (usually a landmark) was identified in each village visited. Using the left hand rule approach, the first household was randomly selected using the date score (combining the two digits of the date, e.g., if research was carried out on the 12^th^, the sum of the two digits of the date was done (1+2=3) and the total sum 3 was taken as the random number for the first household). The interviewers would then use this random number to identify which house to start interviews (e.g., the 3^rd^ house to the left of the starting point). Interviewers would ask if anyone from the target age (18-35) was present; if yes, they would start the screening process. After a successful interview, interviewers would skip 4 households and attempt an interview in the 5^th^ house keeping the left hand rule, and so forth. If there were no eligible participants in the household, interviewers would proceed to the next house until a successful interview was completed; and then subsequently apply the 5^th^ household rule until the quota for that village was met or the day ended.”*

As mentioned in our response to Reviewer 1, we did not collect data on refusals. This has been addressed in the discussion section as a limitation using the following language: “*Another limitation is that we did not collect data on number and nature of refusals, so we cannot comment on the nature of people opting out of this research and how it may have influenced our results.”*

We did collect data for reasons for screen-out. The following language has been added to the results section: *“A total of n=1021 individuals were interviewed; 62 were screened out for various reasons: being under age (n=35), not agreeing to consent (n=3), currently on ART or PrEP (n=11) and being HIV-positive (n=11).”*

*We hope this additional information provides greater clarity for the reader,*
